# Diode Laser Excision of Blandin-Nuhn Mucocele

**DOI:** 10.7759/cureus.7441

**Published:** 2020-03-28

**Authors:** Domenico De Falco, Daniela Di Venere, Eugenio Maiorano

**Affiliations:** 1 Dentistry, University of Bari Aldo Moro, Bari, ITA; 2 Department of Emergency and Organ Transplantation, University of Bari Aldo Moro, Bari, ITA

**Keywords:** minor salivary glands, blandin-nuhn glands, diode laser, oral surgery

## Abstract

The glands of Blandin and Nuhn are mixed mucous and serous glands that are embedded within the musculature of the anterior tongue ventrum. The differential diagnosis for lesions in that area is often challenging and includes other salivary and nonsalivary lesions. This affects decision-making, surgical management, and measures to prevent complications. Unlike conventional cold blade surgery, diode laser use may simplify such treatment.

## Introduction

Blandin and Nuhn mucoceles (BNMs) are rare benign lesions, which develop on the ventral side of the tongue in newborns as well as pediatric and adult patients [1,2]. These lesions are frequently misdiagnosed as conventional mucoceles or ranulas [1-3]. Despite the small size of Blandin and Nuhn glands (8 mm wide and 12-25 mm deep), BNMs can sometimes have large dimensions [4]. Nevertheless, their management with marsupialization is not recommended, leaving surgical excision as the treatment of choice [5,6]. Conventional surgery (scalpel and stitches) may create discomfort, especially in children, as it can involve intraoperative bleeding, postsurgical edema, and related difficulties in chewing and swallowing. Diode laser surgery is characterized by a lack of bleeding during cutting, reduction of postoperative edema, absence of unnecessary stitches, and fast mucosal healing, and therefore represents a superior treatment option for BNMs [7].

## Case presentation

**Figure 1:**
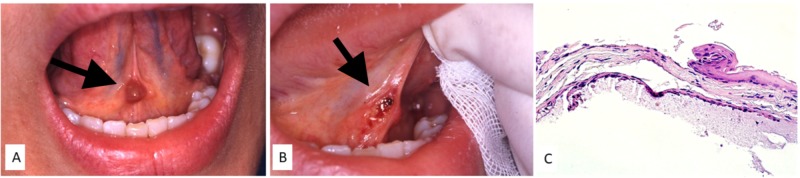
Red-brown lesion of the ventral tongue (A) surgically removed by diose laser without bleeding or unnecessary stitches (B); histological examination showing a thin epithelial lining with foci of extravasation containing mucin (C).

A 28-year-old female was referred to us for the management of a persistent lesion on the floor of the mouth. Intraoral examination showed a red-brown firm, painless, and round lesion of the ventral tongue that had been present for six months (Figure 1A). Diode laser-assisted excision was suggested, and the patient agreed to the procedure. With minimal local infiltration of anesthesia, the lesion was surgically removed using a diode laser with a wavelength of 800 ± 10 nm, in continuous modality, and with an output energy of 1.5 W (Figure 1B); no intraoperative bleeding was observed and stitches were unnecessary. Histological examination revealed a cystic lesion containing mucin with a surrounding epithelial lining, consistent with a diagnosis of BNM (Figure 1C). The surgical wound healed completely in 12 days.

## Discussion

The Blandin-Nuhn glands are a small group of mixed mucous and serous salivary glands, with five to seven small duct openings in the oral cavity, situated on the midline of the ventral tongue [1,2,8]. Although BNMs are commonly described as uncommon lesions, several reports exist in the literature [5]. They typically require invasive surgical management, which may be associated with postoperative complications [2,4,5]. 

The introduction of laser therapy as a treatment option, with reduced bleeding and postoperative edema, absence of unnecessary stitches, and fast mucosal healing, represents a useful innovation in oral surgery such as the excision of benign and malignant neoplasms, surgical or nonsurgical periodontal treatment, decontamination, management of gingival overgrowth, and photocoagulation of oral cavity venous malformations [7,9-12]. As reported in this case, laser diode intervention appears to be less invasive than conventional surgery with cold blade excision and stitches, and it would be particularly preferable in newborns and uncooperative children, with increased acceptability of the procedure by parents.

## Conclusions

Surgical removal of proliferating lesions of the ventral tongue may represent a challenge for the surgeon, especially in uncooperative patients. The highlighted advantages of the surgical removal by diode laser, especially less amount of local anesthesia, rapidity, lack of bleeding and no suture, unlike conventional scalpel surgery, may surely simplify the procedure itself as well improve patient compliance. In addition, the uncomplicated postoperative course with minimal or completely absent pain and edema further improves the overall clinical benefits of diode laser surgery.
